# The influence of a community intervention on influenza vaccination knowledge and behavior among diabetic patients

**DOI:** 10.1186/s12889-019-8101-6

**Published:** 2019-12-27

**Authors:** Lili Tao, Ming Lu, Xiaoning Wang, Xiaoyan Han, Shuming Li, Haiyan Wang

**Affiliations:** 1 Department of Chronic Diseases Prevention and Control, Beijing Chaoyang District Centre for Disease Control and Prevention, No. 25 Panjiayuan Huaweili, Chaoyang District, Beijing, China; 2 Peking Union Medical College, Cancer hospital, No. 17 Panjiayuan Nanli, Chaoyang District, Beijing, China; 3 Beijing Friendship Hospital affiliated to Capital Medical University, Beijing, China

**Keywords:** Diabetic patients, Comprehensive community intervention, Influenza, Vaccination

## Abstract

**Background:**

This study was conducted to evaluate the impact of a comprehensive community intervention on cognition and inoculation behaviors of diabetic patients immunized with influenza vaccine.

**Methods:**

A total of 1538 diabetic patients aged 35 years and above for outpatient visits and follow-up treatments were selected from six community health service centers (three for the experimental group, and the other three for the control group) in Chaoyang District, Beijing. Comprehensive interventions applied to the experimental group include patient intervention and community climate interventions. We compared the total awareness of influenza vaccine knowledge and influenza vaccination rates between the two groups before and after the intervention.

**Results:**

Before the intervention, the total awareness rate of influenza vaccine in the experimental group and the control group was similar (50.6 and 50.2%, respectively. *P* = 0.171). After the intervention, the awareness rate of influenza vaccine in the experimental group and the control group increased. The amplitude of the increase was similar (70.3 and 70.1%, respectively. *P* = 0.822,). Before the intervention, there was no significant difference in the influenza vaccination rate between the experimental group and the control group (29.0 and 26.8%, respectively. *P* = 0.334). After the intervention, the vaccination rate of the experimental group was higher than that of the control group. The difference was statistically significant (The vaccination rate 45.8 and 27.4% for the experimental group and the control group, respectively. *P* < 0.001).

**Conclusion:**

Comprehensive community interventions had a positive effect on vaccination in diabetic patients.

**Trial registration:**

ChiCTR1900025194, registered in Aug,16th, 2019. Retrospectively registered.

## Background

With the improvement of people’s living standards, the prevalence of diabetes has been increasing globally. There are 114 million diabetics in China and the prevalence rate of diabetic patients is 10.9% [1]. In addition, China is also one of the countries with the highest economic burden of diabetes. In 2017, China’s total medical expenditure for diabetes was 63.1 billion U.S. dollars, and the per capita burden was 549.4 U.S. dollars [1].

Because of the existence of metabolic and immune dysfunction, diabetic patients have weaker resistance to bacteria and viruses and are prone to various respiratory infections. Influenza and pneumonia are the most common respiratory infections in diabetic patients. Once infected with the influenza virus, the diabetic patients show increased blood glucose levels, which is difficult to be controlled [2]. The increased blood glucose levels not only aggravate the underlying condition, but also induce toxic pneumonia, secondary bacterial pneumonia, other virus/bacteria co-infection, and increase all-cause mortality [2]. A large number of studies have shown that influenza vaccination is one of the effective preventive measures to prevent and control influenza [3]. A systematic review and meta-analysis of 11 descriptive studies have shown that the risk of all-cause hospitalization in adult patients with diabetes can be reduced by 58% after influenza and pneumonia vaccines are administered, and the risk of hospitalization due to influenza and pneumonia is reduced by 43%. These studies have also shown that the risk of all-cause death in elderly patients with diabetes, the risk of hospitalization and the risk of hospitalization for influenza and pneumonia is reduced by 38, 23 and 45% [4], respectively.

Annual seasonal influenza vaccination can reduce the incidence of influenza significantly, yet influenza vaccination coverage remains low in Asia compared to the West [5]. A total of 30 to 65% of the population in the US receive influenza vaccine each year [6]. However, the influenza vaccination rate in China is less than 2% [7]. From October 2009 to December 2011, the influenza vaccination coverage in diabetics in China was 9.4% [8]. So far, there is no vaccine subsidy programs for diabetic patients in Beijing. This is quite a long way from the proposal by the World Health Assembly in 2013, in which it was proposed that the influenza vaccination rate of the high-risk group should reach 75% by 2010 [9].

In summary, it is of great significance to explore a targeted intervention in China to increase the influenza vaccination rate of diabetic patients. In this study, we conducted comprehensive interventions in the communities to increase awareness among community health workers and diabetics about that vaccine can prevent patients from infectious factors, evaluated the effectiveness of community interventions, and explored the effectiveness of the community-based diabetes management through the use of immunization interventions.

## Methods

### Research object

Voluntary diabetic patients aged 35 years and above for outpatient visits and follow-up treatments were selected from six community health service centers, which were randomly chosen in Chaoyang District, Beijing. Diabetes patients who were not guaranteed to attend follow-up on time were not included in this study.

### Sampling size

According to the sample calculation formula:

$$ {\mathrm{n}}_1={\mathrm{n}}_2=1641.4{\left[\frac{\left({\mathrm{u}}_{\upalpha}+{\mathrm{u}}_{\upbeta}\right)}{\sin^{-1}\sqrt{{\mathrm{p}}_1}-{\sin}^{-1}\sqrt{{\mathrm{p}}_2}}\right]}^2 $$

where, n1 and n2 are the required size of two samples; p1 and p2 are the estimated values of the two overall rates; u_α_and u_β_are the u values corresponding to the test levels α and II error probability β, respectively. According to the data recorded in the literature [4], p_1_ was set to 9.4%, p2 was set to 15%, α was set at 0.05, and β was set at 0.1. The total size of the experimental group and the control group were 719 cases. Considering the loss of follow-up, n_1_ and n_2_ were increased by 10%, and the final sample sizes of both were 800.

This was a cluster randomized trial. The researcher of this subject used random number table to generate the random allocation sequence of community health service centers. A total of 800 voluntary diabetic patients in the experimental group were enrolled from three community health service centers, and 800 diabetic patients were enrolled from three other community health service centers, which were used as the control group. Participants was blinded after assignment to interventions. All intervention and control community health centers can provide influenza vaccination service.

### Intervention and investigation method

In the first step, a baseline survey was conducted in July 2016 on both of the experimental group and the control group by face-to-face interview with study staff. The core content included the demographic data of the objects (age, gender, occupation, educational level, etc.), the awareness of diabetes and influenza vaccine, the situation of past diseases, and the history of influenza vaccination from September 2015 to July 2016.

In the second step, a comprehensive community intervention was conducted by general practitioners (GPs) from August 2016 to January 2017 in the experimental group, including patient intervention and community atmosphere intervention. The patients were followed up one time per month for 6 months. Each intervention included the dissemination of related publicity folds to the group of diabetic patients, the explanation of the vaccine to prevent infectious factors, the knowledge of chronic disease prevention and control, and the core information of influenza vaccination (inoculation significance, appropriate crowd, timing, effect, and adverse reaction). Influenza vaccination should be recommended to patients who have not recently been vaccinated, and a health prescription for influenza vaccination should be issued. GPs provided individually health consultation for influenza vaccination. Community atmosphere intervention refers to the establishment of a related knowledge bulletin board in the community center of the project to provide one diabetes and influenza vaccination session per month for diabetic patients. The total number of sessions were six.

In the third step, a final survey was launched in February 2017. It included awareness of diabetes, awareness of influenza vaccine and pneumococcal vaccine, influenza vaccination during this vaccination season, and reasons of receiving influenza vaccination.

### Calculation

The total awareness rate was calculated as follows,

$$ \mathrm{Total}\ \mathrm{awareness}\ \mathrm{rate}=\frac{\sum \mathrm{The}\ \mathrm{number}\ \mathrm{of}\ \mathrm{questions}\ \mathrm{correctly}\ \mathrm{answered}\ \mathrm{by}\ \mathrm{each}\ \mathrm{of}\ \mathrm{the}\ \mathrm{respondents}}{\mathrm{Number}\ \mathrm{of}\ \mathrm{questionnaires}\ast \mathrm{total}\ \mathrm{number}\ \mathrm{of}\ \mathrm{questions}}\times 100\% $$

### Statistical method

Statistical analysis was carried out by Statistical Product and Service Solutions 20.0 (SPSS). The measurement data were expressed by $\left(\overline{x}\pm s\right)$ and tested by T-test. Enumeration data were expressed by the composition ratio (%) and tested by χ^**2**^. The test level was 0.05.

### Quality control

The questionnaire was conducted by uniformly trained investigators. The authenticity of the questionnaire was verified by quality control personnel. The missing or illogical questionnaires were excluded.

### Role of funding source

This study was supported by Pilot project on chronic diseases and immune prevention policy of China Preventive Medicine Association (20170101). The funding body had no role in the design of the study and collection, analysis, and interpretation of data and in writing the manuscript.

## Results

### Basic situation

A total of 1601 people participated in the baseline survey of this study, including 800 in the experimental group and 801 in the control group. A total of 1538 patients were followed up in the final investigation (31 in the experimental group and 32 in the control group were lost to follow-up), of which, 769 were in the experimental group and the control group. The average age of the subjects in the experimental group was 67.5 years old. There were 225 males (33.2%) and 514 females (66.8%). The majority of the patients were retirees (672, 87.4%). The married/cohabiting patients accounted for 93.8% (721). A total of 708 patients (92.0%) possessed a diploma of junior high school or higher. The average age of the control group was 67.5 years old. There were 298 males (38.8%) and 471 females (61.2%). The majority of the patients were also retirees (701, 91.2%). The married/cohabiting patients accounted for 92.3% (710). A total of 686 patients (89.2%) possessed a diploma of junior high school or higher. There were statistical differences between the experimental group and the control group except for gender and occupation ((${\upchi}_{\mathrm{gender}}^2$ *=*5.221, *P*_*gender*_ = 0.022; ${\upchi}_{\mathrm{occupation}}^2$ =6.070, *P*_*occupation*_ = 0.048)), and there was no statistical difference in other aspects (See Table 1).

**Table 1:** Characteristics of participants at baseline

Items	the experimental group		the control group		χ2	P
	Number(n)	Composition ratio (%)	Number(n)	Composition ratio (%)		
Age						
35~59	161	20.9	175	22.8	6.533	0.088
60~69	299	38.9	264	34.3		
70~79	201	26.1	236	30.7		
80~89	108	14.0	94	12.2		
Gender						
Male	255	33.2	298	38.8	5.221	0.022
Female	514	66.8	471	61.2		
Occupation						
Worker/agriculture/business and service personnel	34	4.4	27	3.5	6.070	0.048
Professional technician / soldier / other	63	8.2	41	5.3		
Retired staff	672	87.4	701	91.2		
Education						
Primary school and below	61	7.9	83	10.8	3.837	0.280
Junior high school graduation	237	30.8	230	29.9		
Senior high school graduation	273	35.5	259	33.7		
College and undergraduate graduation and above	198	25.7	197	25.6		
Marriage						
Married/Cohabiting	721	93.8	710	92.3	1.215	0.270
Other	48	6.2	59	7.7		
Total	769	100.0	769	100.0		

### Comparison of knowledge of influenza vaccine among diabetic patients before and after the intervention

Before the intervention, the total awareness rate of influenza vaccine in the experimental group and the control group was 50.6% (1947/3845) and 50.2% (2007/3845), respectively. There was no significant difference between the two groups (χ^2^ = 1.874, *P* = 0.171). After the intervention, the total awareness rate of influenza vaccine in the experimental group and in the control group was 70.3% (2703/3845) and 70.1% (2694/3845), respectively. Similarly, no significant difference was observed between the two groups (χ^2^ = 0.050, *P* = 0.833). After the intervention, the total awareness rate of influenza vaccine in the experimental group was higher than that before the intervention, and the difference was statistically significant (*P* < 0.001). After the intervention, the total awareness rate of influenza vaccine in the control group also increased, and the difference was statistically significant (*P* < 0.001) (see Table 2).

**Table 2:** Comparison of awareness status of participants on influenza vaccine at two different stages

Period of time	group	Total number of questions	Correct number of questions	the total awareness rate(%)	χ2	P
Before intervention	the experimental group^a^	3845	1947	50.6	1.874	0.171
	the control group^b^	3845	2007	50.2		
After intervention	the experimental group	3845	2703	70.3	0.050	0.822
	the control group	3845	2694	70.1		

These items used to assess the awareness of influenza were from Table 3

^a^The total awareness rate of influenza vaccine in the experimental group before and after the intervention was statistically different.(χ^2^ = 310.916, *P* < 0.001)

^b^The total awareness rate of influenza vaccine in the control group before and after the intervention was statistically different.(χ^2^ = 258.3, *P* < 0.001)

### Awareness of influenza vaccine among diabetic patients before and after the intervention

Before the intervention, 68.4% of the patients in the experimental group thought that the diabetic patients were prone to concomitant infection; 46.6% of the patients believed that flu and pneumonia vaccine could prevent acute complications of diabetes; 49.3% of the patients believed that vaccination against chronic vaccination could reduce the outpatient and hospitalization risk. The answers to the above questions were similar to those of the control group. After the intervention, it was considered that patients with diabetes were more likely to have concurrent infections, flu vaccine could prevent acute complications of diabetes, and the proportion of people vaccinated with flu or/and pneumonia in chronic diseases could reduce the risk of outpatient or/and hospitalization in the experimental group and the control group. The awareness rates were increased in both groups. The results are listed in Table 3.

**Table 3:** Awareness status of participants on influenza vaccine at two different stages

Questionnaire content	Before intervention				After intervention			
	the experimental group		the control group		the experimental group		the control group	
	Number(n)	Composition ratio (%)	Number(n)	Composition ratio (%)	Number(n)	Composition ratio (%)	Number(n)	Composition ratio (%)
Is it easy for diabetics to be complicated with infections?								
Yes	526	68.4	546	71.0	658	85.6	615	80.0
No	54	7.0	86	11.2	35	4.6	82	10.7
Unknown	189	24.6	137	17.8	76	9.9	72	9.4
Will infection worsen the condition and lead to death?								
Yes	491	63.9	520	67.6	607	78.9	626	81.4
No	61	7.9	59	7.7	62	8.1	55	7.2
Unknown	217	28.2	190	24.7	100	13.0	88	11.4
What are the acute complications of diabetes? (multiple choice)								
All right	193	25.1	250	32.5	354	46.0	418	54.4
Wrong/missing	287	37.3	248	32.3	310	40.3	220	28.6
Unknown	289	37.6	271	35.2	105	13.7	131	17.0
Can flu and pneumonia vaccine prevent acute complications of diabetes?								
Yes	358	46.6	300	39.0	550	71.5	466	60.6
No	64	8.3	129	16.8	49	6.4	120	15.6
Unknown	347	45.1	340	44.2	170	22.1	183	23.8
Can vaccination with flu vaccine in chronically ill patients reduce the risk of outpatient and hospitalization?								
Yes	379	49.3	391	50.9	534	69.4	569	74.0
No	88	11.4	45	5.9	47	6.1	50	6.5
Unknown	302	39.3	333	43.3	188	24.5	150	19.5

### Comparison of influenza vaccination status in diabetic patients before and after the intervention

Before the intervention, the number of influenza vaccination in the experimental group and the control group was 223 (29.0%) and 206 (26.8%), respectively. There was no significant difference between the two groups (χ^2^ = 0.934, *P* = 0.334). After the intervention, the number of vaccinations in the experimental group and the control group was 352 (45.8%) and 211 (27.4%), respectively. The difference was statistically significant (χ^2^ = 55.703, *P*<0.001) (see Table 6). In addition, the difference in the influenza vaccination rates between the experimental groups before and after the intervention was also statistically significant (χ^2^ = 43.432, *P*<0.001) (see Table 4).

**Table 4:** Comparison of effects of intervention on influenza vaccination of participants at two different stages

Period of time	Group	Total (n)	Number of vaccinations(n)	Vaccination rates(%)	χ^2^	*P*
Before intervention	the experimental group^a^	769	223	29.0	0.934	0.334
	the control group^b^	769	206	26.8		
After intervention	the experimental group	769	352	45.8	55.703	< 0.001
	the control group	769	211	27.4		

^a^Before and after the intervention, the influenza vaccination rate of the experimental group was statistically different. (χ^2^ = 43.432, *P* < 0.001). There was no significant difference in influenza vaccination rates in the control group after the intervention. (χ^2^ = 0.082, *P* = 0.774)

### Influenza vaccination status of diabetic patients with different characteristics in the experimental group before and after the intervention

According to age stratification, comprehensive interventions had the most significant effect on the vaccination rate of the experimental group in the 35–59 age group compared with other age groups (*OR* = 5.64, *95%CI*: (2.72, 11.70)). The vaccination rate in the above age group was 7.5 and 25.5% before and after the intervention, respectively. If stratified by the education level, the influenza vaccination rate of the experimental group with different education levels increased before and after the intervention, and the increase rate was not statistically different (${\upchi}_{culture}^2$ =3.079, *P*_*gender*_ = 0.380). In addition, the interventions were better for people with good marital status (*OR* = 2.41, *95%CI:* (1.93, 3.00)). Stratified according to the flu vaccination history, the intervention had a greater impact on the patients with no flu vaccination history (*OR* = 8.54, *95%CI*: (5.64, 12.93)) (see Tables 5 and 6).

**Table 5:** Summary of influenza vaccination among population groups with different demographic characteristics

Group	Before intervention				After intervention			
	the experimental group		the control group		the experimental group		the control group	
	Number of vaccinations(n)	Vaccination rates(%)	Number of vaccinations(n)	Vaccination rates(%)	Number of vaccinations(n)	Vaccination rates(%)	Number of vaccinations(n)	Vaccination rates(%)
Age								
35~59	12	7.5	3	1.7	41	25.5	10	5.7
60~69	104	34.8	58	22.0	156	52.2	67	25.4
70~79	61	30.3	110	46.6	99	49.3	102	43.2
80~89	46	42.6	35	37.2	56	51.9	32	34.0
Gender								
Male	87	34.1	74	24.8	117	45.9	80	26.8
Female	136	26.5	132	28.0	235	45.7	131	27.8
Occupation								
Worker/agriculture/business and service personnel	7	20.6	2	7.4	15	44.1	4	14.8
Professional technician / soldier / other	9	14.3	2	4.9	12	19.0	5	12.2
Retired staff	207	30.8	202	28.8	325	48.4	202	28.8
Education								
Primary school and below	26	42.6	33	39.8	33	54.1	31	37.3
Junior high school graduation	69	29.1	48	20.9	110	46.4	50	21.7
Senior high school graduation	55	20.1	57	22.0	104	38.1	60	23.2
College and undergraduate graduation and above	73	36.9	68	34.5	105	53.0	70	35.5
Marriage								
Married/Cohabiting	209	29.0	183	25.8	340	47.2	192	27.0
Other	14	29.2	23	39.0	12	25.0	19	32.2
Total	223	29	206	26.8	352	45.8	211	27.4

**Table 6:** Effects of intervention measures on influenza vaccination among different population groups

Group	the experimental group	the control group		Homogeneity test	
	Number of vaccinations / total number (vaccination rate)(%)	Number of vaccinations / total number (vaccination rate)(%)	*OR* (95%CI)	χ^2^	*P*
Age					
35~59	41/161(25.5%)	10/175(5.7%)	5.6(2.7, 11.7)	18.954	< 0.001
60~69	156/299(52.2%)	67/264(25.4%)	3.2(2.2, 4.6)		
70~79	99/201(49.3%)	102/236(43.2%)	1.3(0.9, 1.9)		
80~89	56/108(51.9%)	32/94(34.0%)	2.1(1.2, 3.7)		
Gender					
Male	117/255(45.9%)	80/298(26.8%)	2.3(1.6, 3.3)	0.060	0.807
Female	235/514(45.7%)	131/471(27.8%)	2.2(1.7, 2.9)		
Occupation					
Worker/agriculture/business and service personnel	15/34(44.1%)	4/27(14.8%)	4.5(1.3, 16.0)	1.410	0.494
Professional technician / soldier / other	12/63(19.0%)	5/41(12.2%)	1.7(0.6, 5.2)		
Retired staff	325/672(48.4%)	202/701(28.8%)	2.3(1.9, 2.9)		
Education					
Primary school and below	33/61(54.1%)	31/83(37.3%)	2.0(1.0, 3.9)	3.079	0.380
Junior high school graduation	110/237(46.4%)	50/230(21.7%)	3.1(2.1, 4.7)		
Senior high school graduation	104/273(38.1%)	60/259(23.2%)	2.0(1.4, 3.0)		
College and undergraduate graduation and above	105/198(53.0%)	70/197(35.5%)	2.0 (1.4, 3.1)		
Marriage					
Married/Cohabiting	340/721(47.2%)	192/710(27.0%)	2.4(1.9, 3.0)	7.848	0.005
Other	12/48(25.0%)	19/59(32.2%)	0.7(0.3, 1.6)		
Influenza vaccination history					
Yes	179/223(80.3%)	182/206(88.3%)	0.5(0.3, 0.9)	72.906	< 0.001
No	173/546(31.7%)	29/563(5.5%)	8.5(5.6,12.9)		

## Discussion

Vaccination against influenza is one of the effective preventive measures to prevent and control influenza [2]. Diabetes patients are at high risk of influenza. The rate of influenza vaccination is low among diabetic patients in China [8]. There is a need for a targeted intervention to increase the rate of influenza vaccination in people with diabetes, and there are currently few studies in this area.

Before and after the implementation of comprehensive intervention measures in the experimental group, the total awareness rate of influenza vaccine knowledge in the experimental group was 50.6 and 70.3%, respectively; the total awareness rate in the control group was 50.2 and 70.1%, respectively. This indicated that the intervention did not significantly promote the knowledge of cognitive influenza in the experimental group. The possible reason was that all community health service centers (where experimental and control groups can be served) in Chaoyang District of Beijing had enhanced their knowledge on the prevention and control of diabetes and the benefits of vaccination against chronic diseases with the advancement of prevention and control of chronic diseases. We conducted community atmosphere intervention on the experimental group in this study, including the production of knowledge bulletin boards, and regular knowledge lectures. All that were the same as the daily chronic disease prevention and control in the community health service centers where the control group got service. The community atmosphere intervention in the experimental group was not well performed. Compared with the control group, the communities in the experimental group did not take more interventions to publicize the knowledge of the influenza vaccine. All of these results might lead to an increase in the total awareness rate of influenza vaccine in the experimental group and the control group after the intervention in this study. However, no statistical difference was observed between the two groups.

Although the total awareness rate of both groups increased and the increase was the same, the influenza vaccination rate (45.8%) of the experimental group after the intervention increased by 18.3% compared with the control group (27.4%). The interventions also included patient intervention implemented in the experimental group in this study, in addition to the community atmosphere intervention. This suggested that GPs played an active role in promoting the vaccination of people with diabetes. Consistent results were also obtained in Altay’s report [10]. In this report, after health education was given to elderly diabetic patients about “the need for vaccination for elderly diabetic patients,” Altay and other scholars found that the flu vaccination rate among elderly diabetic patients after education (36.6%) increased compared with that pre-education (12.1%) [10]. Liao’s study [11] found that highlighting disease consequences becomes increasingly important when its influenza case-fatality ratio (CFR) increases, for promoting vaccination uptake. A meta-analysis in mainland China [12] showed that in the analysis of influencing factors, those recommended by healthcare workers was the most reported reasons for influenza vaccination.

In addition, our study considered that the uneven distribution of gender and occupational factors between the experimental group and the control group may cause a shift in the results, and a subgroup analysis method was adopted. The results showed (see Table 6) that in different genders and occupational groups, the effect of interventions on influenza vaccination was not heterogeneous. Hence, the uneven distribution of gender and occupation between the two groups did not change the conclusion.

It should be mentioned that the study population may not represent the general population of China, because their mean age were 67.5 years old and most of them were retired staffs, and one quarter of subjects had more than college degrees. And part of the intervention effect may have been the result of a Hawthorne effect.

The total awareness rate of the control group was increased, and the vaccination rate did not change significantly. The possible reason was that the control group lacked flu vaccination guidance recommendation from the GPs. One of the epidemiological surveys showed that “no one recommended flu vaccine” is a more common cause of none flu vaccination in high-risk populations [13]. In another survey, 78% of respondents over the age of 60 are willing to follow the GPs’ advice on flu vaccination [14]. Although people with diabetes increase their awareness of flu vaccine through community propaganda, this does not mean they are willing to get a flu vaccination. When they directly accepted the vaccination advice from the GPs, they would choose to get the flu vaccination. In summary, in order to promote influenza vaccination for diabetic patients, we should focus on strengthening the training of flu-related knowledge of community GPs, and then they would promote the benefits of flu vaccine to diabetic patients and give flu vaccination prescription.

In this study, it showed that the rate of influenza vaccination in the 35–59 age group both in the intervention group (25.5%) and the control group (5.7%) was lower than the other age groups (the intervention group: 60–69 (52.2%), 70–79 (49.3%), and 80–89 (51.9%), the control group: 60–69 (25.4%), 70–79 (43.2%), and 80–89 (34%)). The possible reason was that the policy of vaccinating influenza vaccines for older people over 60 years of age in Beijing from 2007 [15] had led to the increase of the influenza vaccination rates for above 60-year-old.

There are several unique features worth mentioning in our study. First of all, the idea and perspective of the study is new. It is the first time to carry out community comprehensive intervention on cognition and inoculation behaviors of diabetic patients with influenza vaccine in China. Secondly, the GPs-based interventions recommended in this study are highly implementable and effective.

This study had several limitations. It lacked a set of criteria for evaluating the quality of community atmosphere intervention. And the influenza vaccination status was self-reported without confirmation from local immunization record system.

## Conclusion

In our study, the vaccination rate of the experimental group was higher than that of the control group after community comprehensive interventions. The difference was statistically significant. In summary, it shows community comprehensive interventions had a positive effect on vaccination in diabetic patients.

## Supplementary information

**Additional file 1:** A brochure for influenza vaccination.

## Data Availability

The datasets generated and/or analysed during the current study are not publicly available due to private protection but are available from the corresponding author on reasonable request.

## Abbreviations

GPs: General Practitioners
SPSS: Statistical Product and Service Solutions 20.0
