# Supplementary figures and images for: The influence of a community intervention on influenza vaccination knowledge and behavior among diabetic patients
Source: BMC Public Health. 2019 Dec 27;19:1747. doi: 10.1186/s12889-019-8101-6 (PMC6935125; doi:10.1186/s12889-019-8101-6)

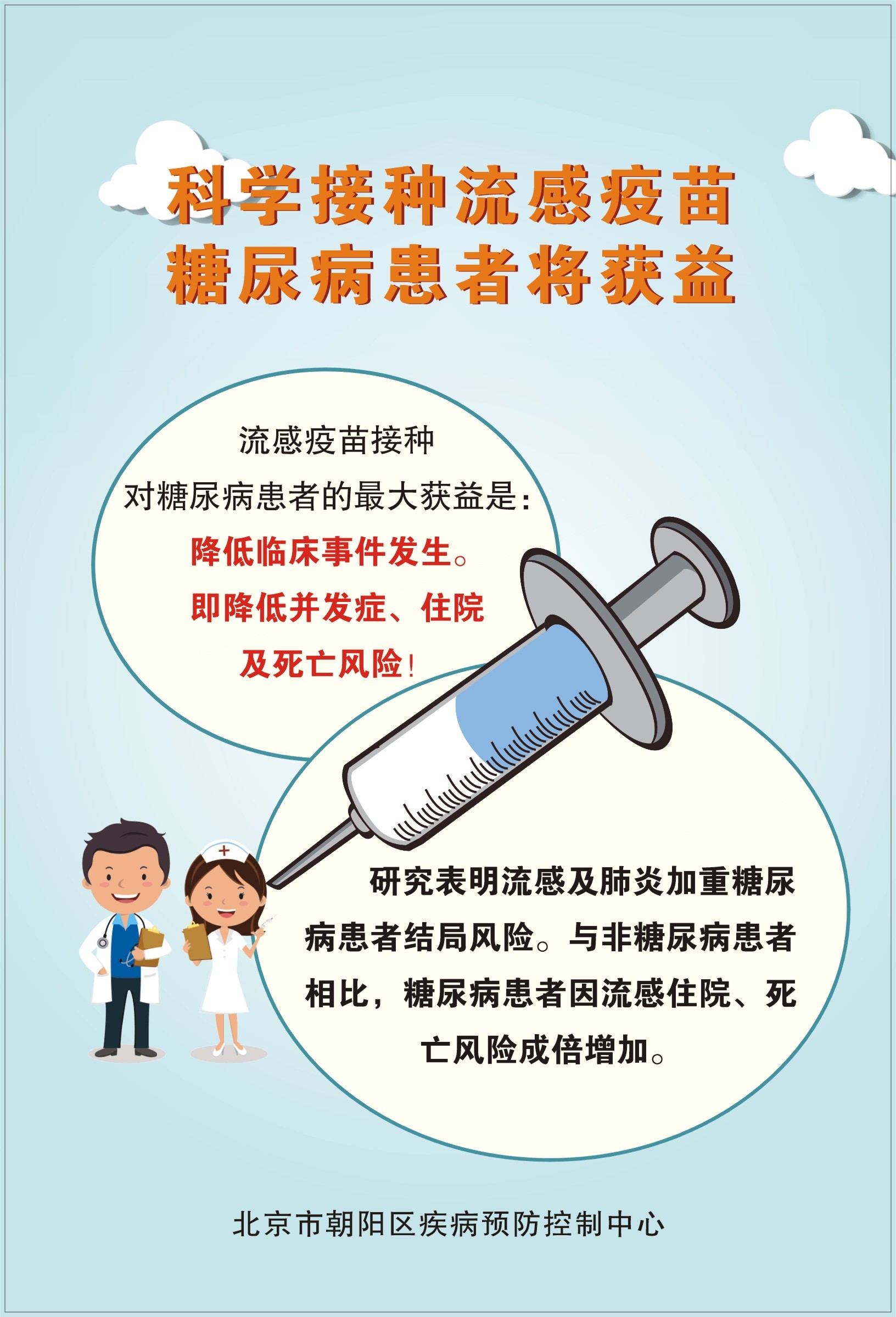

Supplement: Supplementary file 1 — Additional file 1: A brochure for influenza vaccination. [file 12889_2019_8101_MOESM1_ESM.jpg]
